# Energy transfer from phycobilisomes to photosystem I at 77 K

**DOI:** 10.3389/fpls.2023.1293813

**Published:** 2023-11-22

**Authors:** Ivo H. M. van Stokkum, Parveen Akhtar, Avratanu Biswas, Petar H. Lambrev

**Affiliations:** ^1^ Department of Physics and Astronomy and LaserLaB, Faculty of Science, Vrije Universiteit Amsterdam, Amsterdam, Netherlands; ^2^ Institute of Plant Biology, HUN-REN Biological Research Centre, Szeged, Hungary; ^3^ Doctoral School of Biology, University of Szeged, Szeged, Hungary

**Keywords:** photosynthesis, time-resolved fluorescence, cyanobacteria, thylakoid membranes, photosystems, *Synechocystis* sp. PCC 6803

## Abstract

Phycobilisomes serve as a light-harvesting antenna of both photosystem I (PSI) and II (PSII) in cyanobacteria, yet direct energy transfer from phycobilisomes to PSI is not well documented. Here we recorded picosecond time-resolved fluorescence at wavelengths of 605–760 nm in isolated photosystem I (PSI), phycobilisomes and intact cells of a PSII-deficient mutant of *Synechocystis* sp. PCC 6803 at 77 K to study excitation energy transfer and trapping. By means of a simultaneous target analysis of the kinetics of isolated complexes and whole cells, the pathways and dynamics of energy transfer *in vitro* and *in vivo* were established. We establish that the timescale of the slowest equilibration between different terminal emitters in the phycobilisome is ≈800 ps. It was estimated that the terminal emitter in about 40% of the phycobilisomes transfers its energy with a rate constant of 42 ns^−1^ to PSI. This energy transfer rate is higher than the rates of equilibration within the phycobilisome – between the rods and the core or between the core cylinders – and is evidence for the existence of specific phycobilisome-PSI interactions. The rest of the phycobilisomes remain unconnected or slowly transferring energy to PSI.

## Introduction

**Figure 1:**
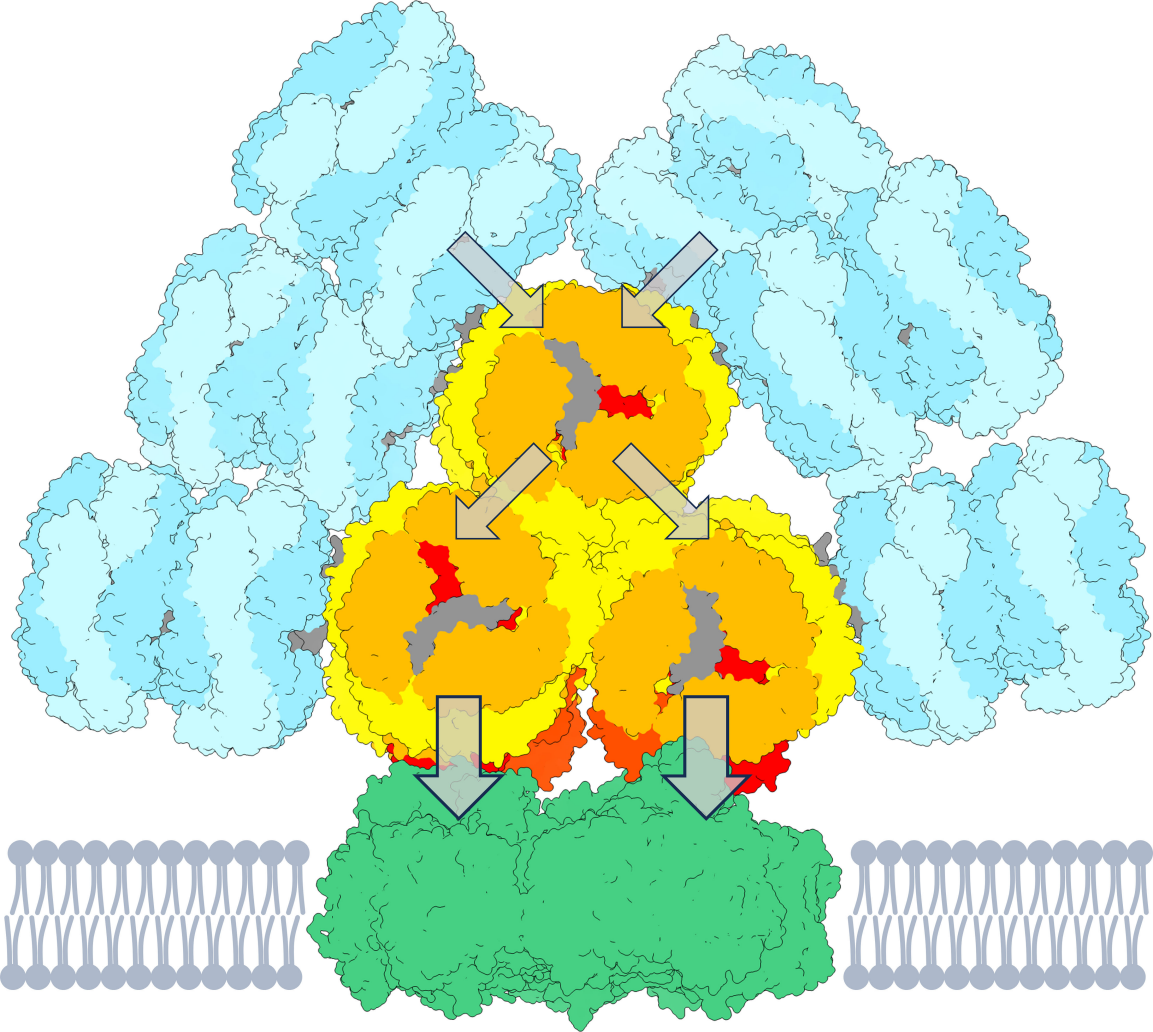
Cartoon representation of a PB-PSI complex. The PB structure (Zheng et al., 2021) consists of rods (cyan) containing PC640 and PC650. The three-cylindrical APC core (yellow–orange) contains APC660 and the red-shifted emitters APC680, presumably located in the subunits ApcD, ApcE, ApcF (red and orange-red). The arrows indicate the flow of energy towards the PSI trimer (green, PDB 5oy0, Malavath et al., 2018). The figure is created using UCSF ChimeraX (Goddard et al., 2018). The pigment stoichiometry is detailed in **Figure S1**.

In cyanobacteria and red algae, the primary light-harvesting function is performed by phycobilisomes (PBs), large extrinsic pigment-protein complexes bound to the cytoplasmic surface of the thylakoid membrane (Glazer, 1984). Structurally, PBs are composed of phycobiliproteins (PBPs), containing phycobilins as colored pigments and colorless linker proteins. The PBs are typically organized in a hemispherical structure with multiple units of the PBPs phycocyanin (PC) and allophycocyanin (APC) (**Figure 1**; **Figure S1**). The latter constitutes the central core that serves as a scaffold for the attachment of the PC rods extending outward. The rods are made up of stacked discs with each disc containing six α/β PC heterodimers and linker proteins. The core typically consists of three cylinders, each containing two stacked APC discs. The bottom two cylinders facing the thylakoid membrane contain the core-membrane linker L_CM_ (ApcE) as well as the APC forms ApcD and ApcF. There are, however, large variations in the PBs organization and composition and deviations from this fundamental design depending on the species and growth conditions. PBs efficiently absorb green-orange light complementing the absorption of chlorophylls (Chls) and carotenoids (Bar-Eyal et al., 2018; Harris et al., 2018). The organization of PBPs into rod-like structures and their close arrangement within the PBs facilitate efficient energy transfer through strong pigment-pigment interactions.

Understanding the connectivity and efficiency of energy transfer is crucial for optimizing the light-harvesting capabilities of photosynthetic organisms. Despite extensive research, the dynamics of energy transfer within the PBs, as well as between the PBs and the two photosystems (PSI and PSII), have been the subject of ongoing debate. PBs efficiently transfer energy to both, PSI and PSII. This is evident from low-temperature fluorescence spectra showing significant emission peaks from both photosystems when PBs are excited (Bruce and Biggins, 1985; Ashby and Mullineaux, 1999). Additional techniques, such as action spectra for flash-induced P_700_ photooxidation at room-temperature, provide further evidence indicating the transfer of energy from phycobilins to PSI (Mullineaux, 1992). PBs can transfer energy to PSI either directly via interaction between the PB and PSI (Su et al., 1992; Mullineaux, 1994; Liu et al., 2013) or indirectly via “spillover” from PSII to PSI (Mcconnell et al., 2002; Ueno et al., 2017). Different subunits of APC are shown to be responsible for energy transfer to either of the photosystems. ApcE is responsible for energy transfer to PSII, whereas ApcD is proposed to serve as an energy donor primarily for PSI (Ashby and Mullineaux, 1999; Dong et al., 2009; Liu and Blankenship, 2019). Furthermore, picosecond fluorescence measurements suggest a relatively slow energy transfer step from the PB core to reaction center Chls, with some PBs decoupled from the reaction centers (Mullineaux and Holzwarth, 1991). These findings highlight the complexity of energy transfer in PBs-photosystems interactions.

Energy transfer from PBs to PSI was also shown in *Anabaena* heterocysts where PSII is absent (Peterson et al., 1981). Using absorption and fluorescence spectral imaging, the PC-rich heterocysts of *Rivularia* were reported to have connections between PSI and PC (Nozue et al., 2017). Furthermore, in *Synechocystis* and other cyanobacterial species, the existence of an alternative PB containing a single PC rod connected to the linker protein CpcL (CpcG2 in Synechocystis) but no APC was reported (Kondo et al., 2005; Mullineaux, 2008). The CpcL-type PBs especially interact with PSI transferring energy directly to it (Kondo et al., 2007; Watanabe et al., 2014). The recent cryo-electron microscopy structure and spectroscopic investigation of a CpcL-type PBs also confirms their connectivity to PSI (Zheng et al., 2023).

Energy transfer pathways have been studied in great detail in isolated systems (PSI, PSII, PBs) as well as intact cells by using different time-resolved spectroscopy techniques and there are several models describing the kinetics in great detail (Tian et al., 2011; Tian et al., 2012). Despite the abundance of experimental data and models, there are contradicting views about the kinetics and rate-limiting steps of energy transfer within PBs and between the photosystems and PBs. The organization and function of the cyanobacterial light-harvesting complexes and reaction centers can be tested using molecular genetics approaches. Based upon the fluorescence kinetics of wild-type *Synechocystis* sp. PCC 6803 and of mutants lacking entirely the PB antenna or parts of it, Van Stokkum et al. (2018) proposed a detailed functional compartmental model of energy transfer. According to the model, the effective trapping time of energy captured by the PBs – about 100–140 ps – is contributed mainly by excitation equilibration within the PBs – in the PC rods and between the rods and the APC core, whereas energy transfer from the lowest-energy pigments in the APC core to Chls in the photosystems is significantly faster. The timescale of energy transfer between the terminal emitter in the PBs and PSII was determined to be about 20 ps based on experiments with a *Synechocystis* sp. PCC 6803 mutant which lacks PSI (Shen et al., 1993; Acuña et al., 2018).

Several deletion mutants of the model cyanobacterium *Synechocystis* sp. PCC 6803 which lack major PSII subunits (D1/D2, CP43, CP47) have been constructed and tested in the laboratory of Dr. W. Vermaas (Yu and Vermaas, 1990; Bittersmann and Vermaas, 1991). In these mutants, PBs stay bound in the intact form to the photosynthetic membrane and energy transfer within the PBs is not affected by the absence of PSII. When compared with wild-type, the mutants showed higher PB fluorescence yield, but considerably smaller than observed in isolated PBs. The difference compared to isolated PBs was proposed to be because of the change in the conformation of PBs *in vivo* and *in vitro* (Bittersmann and Vermaas, 1991). Using 77 K fluorescence emission spectroscopy in combination with microsecond flash-induced P700 photooxidation, Mullineaux (1994) reported that in PSII deficient cells, PBs transfer energy to PSI with a quantum efficiency of about 80%, compared to 40% in the wild-type. However, no studies have been reported that directly resolve the kinetics of excitation energy transfer (EET) from PBs to PSI.

In this study, we investigated energy transfer at 77 K in PBs and PSI *in vitro* and *in vivo* in a mutant of *Synechocystis* sp. PCC 6803 completely lacking PSII due to a deletion of the *psbA* (D1), *psbC* (CP43), and *psbD* (D2) genes. WT and the PSII-deficient mutant were cultured under similar conditions. The dynamics of energy transfer were measured in isolated PB and PSI complexes (WT) and in intact cells of both the WT and PSII-deficient mutant using time-correlated single photon counting, selectively exciting either PBs or Chls in PSI/PSII. A detailed kinetic model was constructed and refined by fitting to the fluorescence kinetics recorded at 77 K. We establish that PBs transfer energy to PSI on two different timescales. What is particularly challenging at 77 K is that a large part of the emission originates from the different pools of Red Chl *a* which are present in PSI (Gobets et al., 2001; Snellenburg et al., 2013). Thus, the modelling also requires a detailed description of the kinetics of the PSI Red Chl *a*.

## Materials and methods

### Growth conditions

The cyanobacterial strains used in this study are *Synechocystis* sp. PCC6803 and a PSII-deficient mutant (hereafter called ΔPSII). The ΔPSII mutant is constructed by deleting the *psbA, psbC* and *psbD* genes and practically lacks all proteins of PSII including D1, D2, CP43 and CP47 (Yu and Vermaas, 1990). WT cells were grown photoautotrophically in BG11 medium supplemented with 5 mM HEPES–NaOH (pH 7.5). For the mutant cells, the medium was supplemented with 10 mM glucose, 40 µg/ml spectinomycin and 8 µg/ml chloramphenicol. The cultures were placed on a rotary shaker (100 rpm) at 30°C under continuous white light (~ 35 μmol photons m^−2^ s^−1^).

### Photosystem I and phycobilisomes preparation

One-week old cells were used for both PSI and PBs isolation. PSI was isolated following the protocol described in Akhtar et al. (Akhtar et al., 2021) by detergent solubilization of freshly isolated thylakoid membranes with 2% n-dodecyl β-D-maltoside (β-DDM) and sucrose density gradient ultracentrifugation. The preparation of PBs was carried out following the protocol described by Garnier et al. (1994) with some modifications described by Akhtar et al. (2022).

### Steady-state fluorescence spectroscopy

Fluorescence emission spectra in the visible range were measured at 77 K using a FP-8500 (Jasco, Japan) spectrofluorometer. The sample was diluted to an absorbance of 0.1 per cm at the red maximum cooled in a home-built accessory used with the FP-8500 spectrofluorometer. Emission spectra in the range of 620–780 nm were recorded with excitation wavelength of 440 nm and 580 nm and excitation/emission bandwidth of 2.5 nm. The measurements were performed with 1 nm increment and 4 s integration time. The spectra were corrected for the spectral sensitivity of the instrument using a calibrated light source (ESC-842, Jasco) as a reference.

### Time-resolved fluorescence spectroscopy

Picosecond time-resolved fluorescence measurements were performed with a time-correlated single-photon counting (TCSPC) instrument (FluoTime 200/PicoHarp 300 spectrometer, PicoQuant). Excitation pulses were provided by Fianium WhiteLase Micro (NKT Photonics, UK) supercontinuum laser with a repetition rate of 20 MHz. Excitation wavelengths of 440 and 580 nm were used to excite selectively Chls and PBs. The cell suspension with the optical density equivalent to 0.03 was placed in a 1 mm demountable cryogenic quartz cell and cooled in an optical cryostat (Optistat DN, Oxford Instruments, UK). Then, the fluorescence decays were recorded at wavelengths of 605–760 nm with 5 nm steps. The total instrument response function (IRF) measured using 1% Ludox as scattering solution has full width at half maximum (FWHM) of ≈40 and ≈50 ps with 440 and 580 nm excitation respectively. The data are corrected for the spectral response of the detector. Global multiexponential lifetime analysis with IRF reconvolution was performed using MATLAB. Target analysis was performed according to Van Stokkum et al. (2004); Van Stokkum et al. (2018) using an equal-area constraint of the SAS to estimate the equilibria (Snellenburg et al., 2013).

## Results and discussion

### Steady-state fluorescence spectroscopy of intact cells

**Figure 2:**
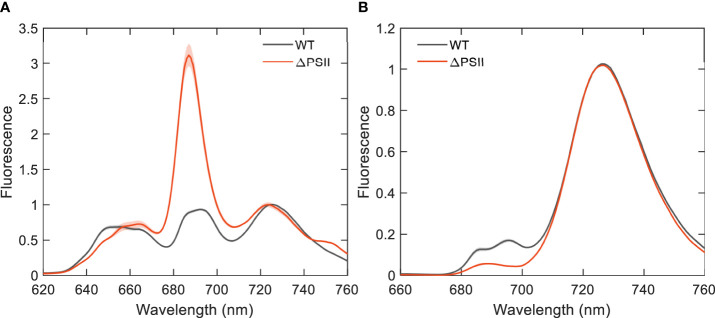
Fluorescence emission spectra of intact cells of *Synechocystis* WT and ΔPSII recorded at 77 K and normalized to the maximum at 724 nm. **(A)** Excitation wavelength 580 nm; **(B)** Excitation wavelength 440 nm. The WT spectra are an average of six and the ΔPSII spectra are an average of four independent experiments; the shaded area represents the standard error.

One of the main goals of this study was to estimate the direct energy transfer from PBs to PSI in intact cells of *Synechocystis* sp. PCC 6803 in the absence of PSII. For this, we used a mutant strain devoid of PSII. As a first approximation, we recorded fluorescence emission spectra at 77 K by selectively exciting either PB or Chl *a* (**Figure 2**). The emission spectra of WT recorded with either excitation are very similar to previously published results (Akhtar et al., 2022). Briefly, the spectra show peaks at 650 and 660 nm corresponding to PC650 and APC660 and peaks at 686/694 and 724 nm – primarily attributed to PSII and the Red Chl *a* of PSI, respectively. The ΔPSII mutant shows strong APC680 emission which indicates that not all energy is transferred from PB to PSI. The fluorescence spectra recorded with 440 nm excitation (almost exclusively absorbed by the photosystems) clearly show missing PSII peaks – 685 nm and 695 nm in the mutant strain, without any change in the PSI emission.

### Global analysis of the fluorescence kinetics of intact cells

**Figure 3:**
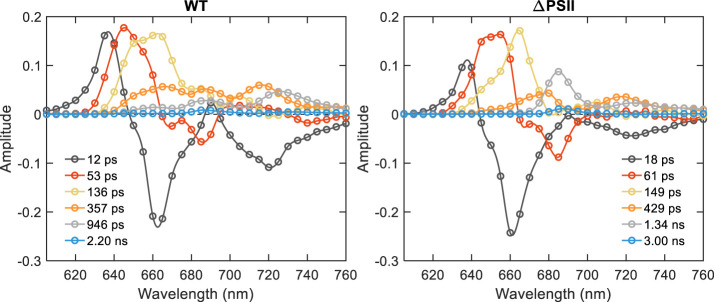
DAS of *Synechocystis* WT and ΔPSII obtained from global analysis of fluorescence decays measured by TCSPC at 77 K with PB (580 nm) excitation.

Picosecond time-resolved fluorescence measurements were performed using TCSPC at 77 K to resolve the dynamics of EET from PB to both photosystems in the intact cells. At 77 K, the emission from the Red Chl *a* of PSI is enhanced – thus allowing better comparison of the two photosystems. The excitation was set to 580 nm and the decay curves were recorded from 605–760 nm to cover both the PB and the Red Chl *a* region. Six lifetimes were necessary to fit the fluorescence decays over a 4-ns time range (**Figure 3**). The lifetimes and DAS of WT are very similar to those previously published (Akhtar et al., 2022). Briefly, the first component with the lifetime of 12 ps shows both energy transfer within the PB and from bulk to Red Chl *a* in PSI (690 to 720 nm). The second component with the lifetime of 53 ps having positive and negative peaks in the DAS can be ascribed to the decay of PC650 and APC660 and the rise of the red-shifted APC680. The 136-ps component with only positive peaks shows decay of all PB components (PC, APC662 and APC680, as the energy is transferred to the Chls of the photosystems). The 357 and 946 ps components correspond to the decay of PSII- and PSI-bound Chls.

Compared to WT, the DAS of ΔPSII showed significant differences. Remarkable are the kinetic differences in the 640–680 nm wavelength range reflecting PB emission - PC emission decays faster than in WT – compare the peaks at 650 nm in the second and third DAS. The third DAS has only one peak at 665 nm, indicating decay of APC. The long-lived DAS with the lifetimes of 429 and 1.34 ns having peaks at 680, 685, 720 and 725 nm are associated with decay of APC680 and Red Chl *a* in PSI.

**Figure 4:**
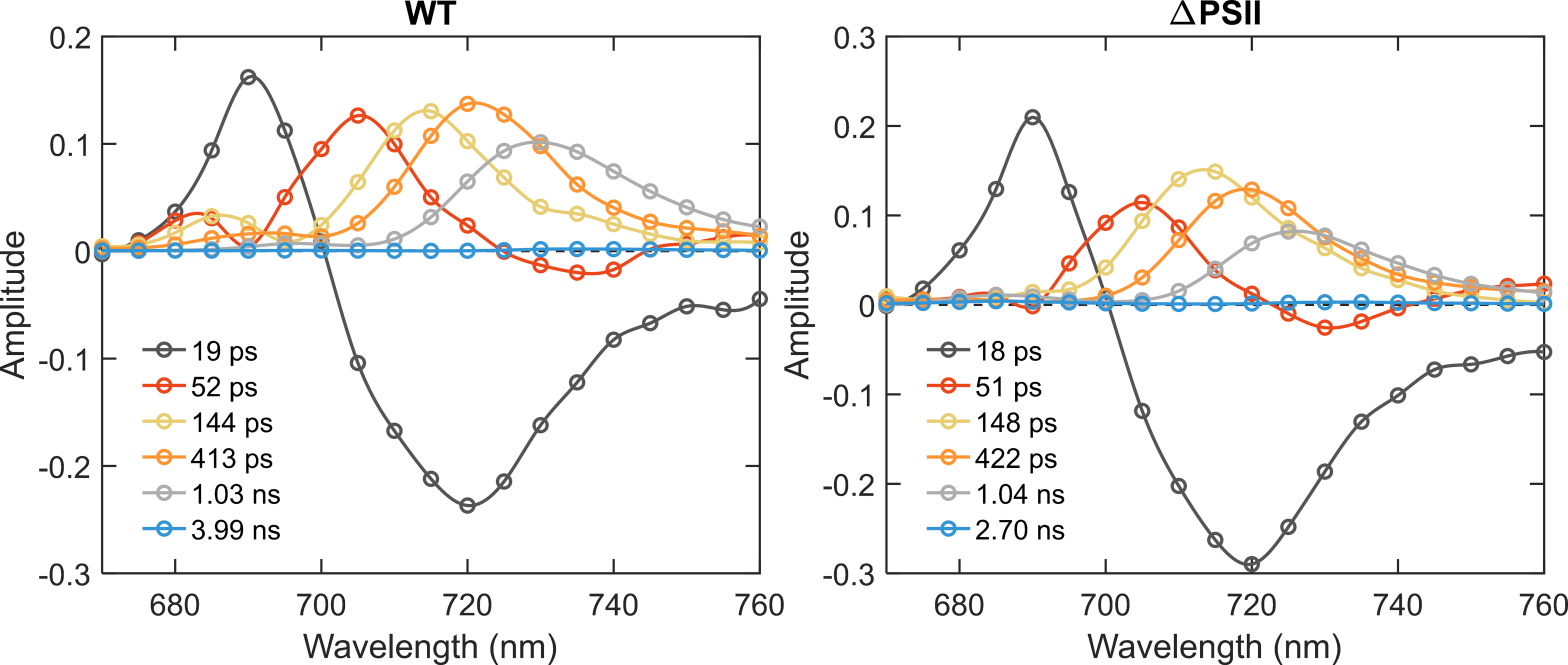
DAS of *Synechocystis* WT and ΔPSII obtained from global analysis of fluorescence decays measured by TCSPC at 77 K with Chl *a* (440 nm) excitation.

**Figure 4** shows the DAS estimated with 440 nm excitation, predominantly absorbed by Chl *a* in the photosystems. The first two components (19 and 52 ps) show energy transfer from Bulk to Red Chl *a* - from 690 nm to 700–710 nm and from 700 nm to 715–725 nm. The components with lifetimes of ≈145 ps, ≈420 ps and ≈1.0 ns have all-positive DAS with maxima around 710, 716, and 722 nm, respectively. No meaningful differences in the fluorescence kinetics or spectra were detected between the WT and ΔPSII apart from changes in the DAS of the short-lived components (52, 145 and 420 ps) below 700 nm, which evidently show PSII emission in WT. These results essentially confirm that the kinetics of PSI are unaffected by the mutation.

### Target analysis of the trimeric PSI complex at 77 K

**Figure 5:**
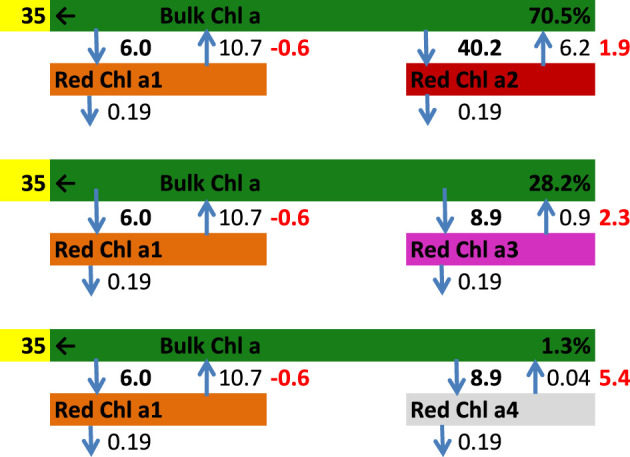
Minimal kinetic scheme of the PSI complex at 77 K with rates in ns^-1^. The dark green compartment represents the Bulk PSI Chl *a* including the reaction center. Four pools of Red Chl *a* have been resolved: brown, maroon, purple and grey. The free energy differences relative to the bulk PSI Chl *a* are indicated in red (in units of k_B_T). The rate of trapping is yellow highlighted.

**Figure 6:**
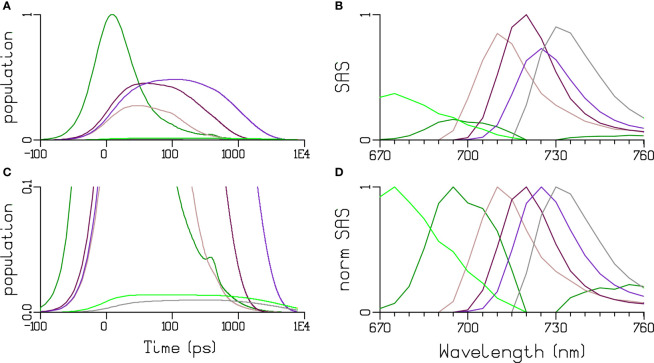
Target analysis of the trimeric PSI complex at 77 K. **(A**, zoom in **C)** Concentrations estimated after 440 nm exc. **(B, D)** SAS and normalized SAS. Key: PSI Chl *a* (dark green). Four pools of red Chl *a* have been resolved in PSI: brown, maroon, purple and grey. Note that the time axis in **(A, C)** is linear until 100 ps and logarithmic thereafter. Light green is a long-lived impurity.

Toward modelling the kinetics of PB-PSI EET in cells, we first performed target analysis of the EET kinetics of the trimeric PSI complex from *Synechocystis* sp. PCC 6803 at 77 K using the high-dynamic range TCSPC data published recently (Akhtar et al., 2021). The signal to noise ratio (SNR) of these TCSPC data is ≈1000 (**Figure S2**) and the width (FWHM) of the IRF is 48 ps. The minimal kinetic scheme of the PSI complex at 77 K consists of three compartments: Bulk PSI Chl *a* including the reaction center, and two pools of Red Chl *a* (**Figure 5**, top%) – a1 and a2. The decay rate constant of 35 ns^−1^ signifies trapping by the reaction center, whereas the Red Chl *a* compartments have a decay rate constant of 0.19 ns^−1^ and are connected to the Bulk Chl *a* with reversible EET. Thus, the Red Chl *a* excited states can be photochemically trapped after uphill energy transfer. This three-compartment system accounts for 70.5% of the PSI complexes and is complemented by two more fractions (28.2 and 1.3%), wherein the energy of the lowest Red Chl *a* is allowed to vary – this is to account for energy disorder. The total model has five distinct types of emitting species – Bulk Chl *a* and four different Red Chl *a* with respective species-associated emission spectra (SAS). The SAS and the species time-dependent populations are plotted in **Figure 6**. An additional long-lived component depicted in light-green indicates an impurity of a very small concentration, cf. **Figure 6C**, possibly a small amount of free Chl *a* with maximum emission at 675 nm (**Figure 6D**), decaying with a 5.2 ns lifetime.

The quality of the target analysis fit is excellent (**Figure S2**). The estimated dynamics of the Red Chl *a* results in free energies of the Red Chl *a* relative to the Bulk PSI Chl *a* of 0.6, -1.9, -2.3 and -5.4 k_B_T. The entropy difference between the Bulk Chl *a* and a pool of Red Chl *a* (assuming it contains 2 pigments) is ≈3.8 k_B_T. Thus, the enthalpy difference ranges from 3.2 to 9.2 k_B_T. This is equivalent to a red shift relative to Bulk Chl *a* of 8-24 nm, which is comparable to the high-energy tail of the estimated Red Chl *a* SAS (Croce et al., 2007; Snellenburg et al., 2013). Thus, the dynamics of trapping after uphill energy transfer is consistent with the gradually increasing Gibbs free energy difference and the increasing red shift of the Red Chl *a* SAS.

### Global and target analysis of PB at 77 K

**Figure 7:**
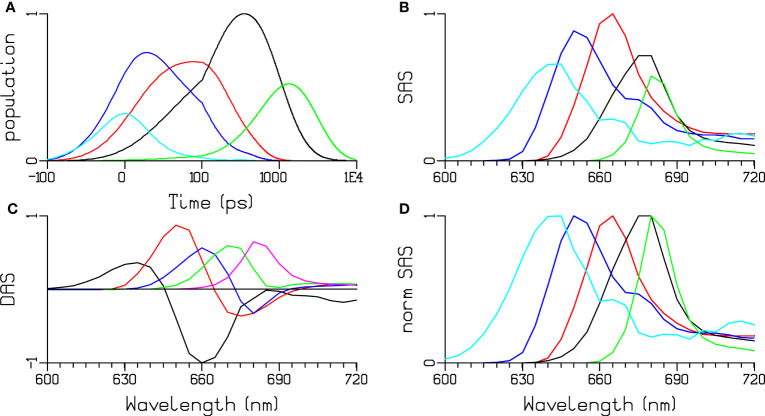
Global and target analysis of PB at 77 K. **(A)** Concentrations estimated after 580 nm exc. **(B, D)** SAS and normalized SAS. Key: PC640 (cyan), PC650 (blue), all APC660 (red), and “APC680” (black and green). Note that the time axis in **(A)** is linear until 100 ps and logarithmic thereafter. **(C)** DAS estimated from the global analysis, key: black 27 ps, red 73 ps, blue 281 ps, green 797 ps, magenta 1.9 ns.

Next, we present the global and the target analysis of the high-quality EET kinetics in isolated WT PB of *Synechocystis* sp. PCC 6803 at 77 K (**Figure 7**). From the global analysis five components have been estimated with lifetimes of 27 ps (black DAS in **Figure 7C**), 73 ps (red), 281 ps (blue), 797 ps (green), and 1.9 ns (magenta). The DAS are consistent with successive downhill energy transfer to the lowest energy APC680 pigments (green SAS in **Figures 7B, D**).

**Figure 8:**
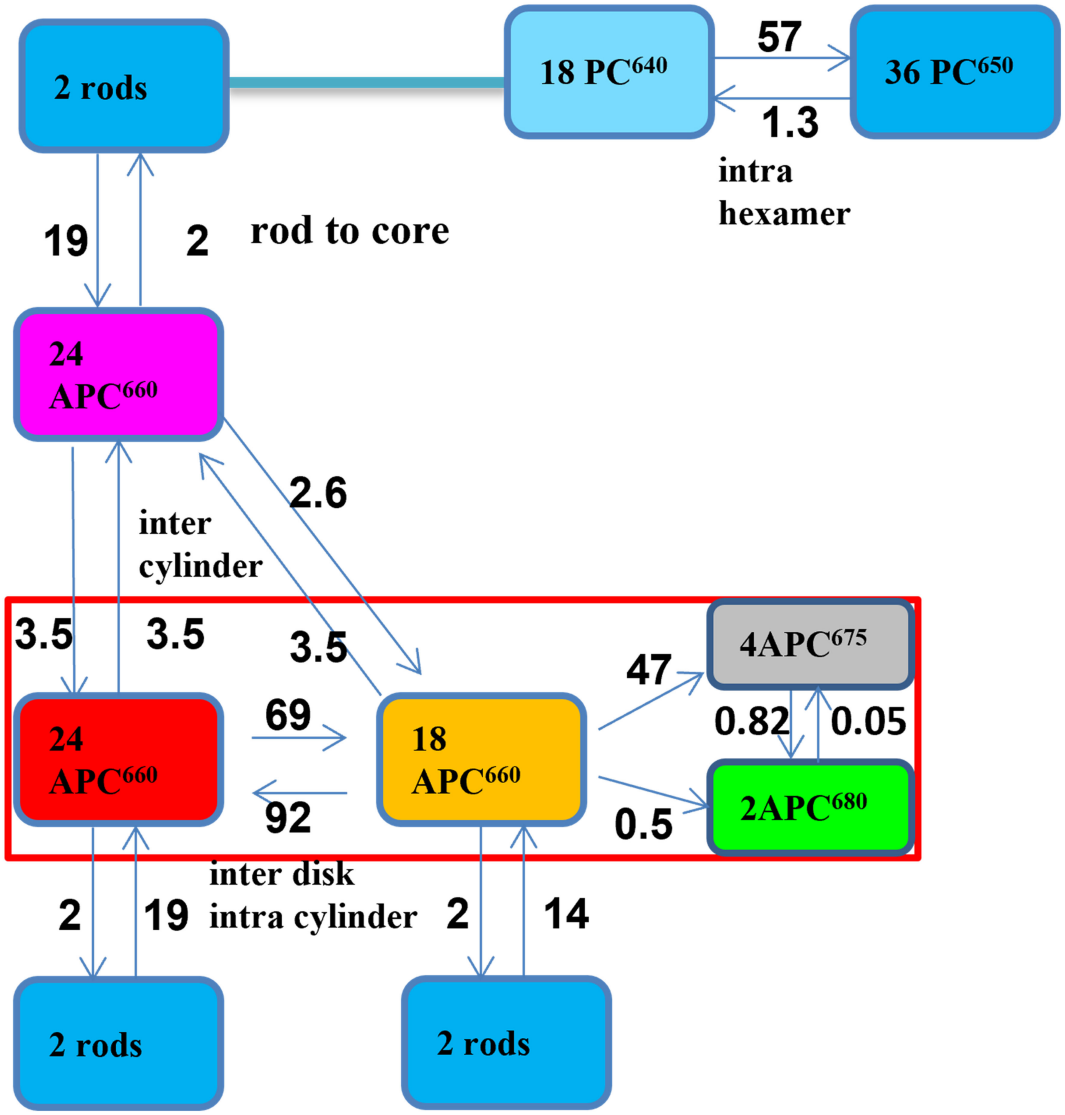
Functional compartmental model of PB at 77 K with microscopic rate constants in ns^-1^, with a zoom out of a rod consisting of three lumped hexamers in the upper right. The rods contain PC640 (cyan) and PC650 (blue). The magenta APC660 compartment represents the top cylinder. The red rectangle indicates the two basal cylinders, with APC660 (orange) and APC680 (black and green) in four discs, and APC660 (red) in four other discs. The common decay rate constant for all excited PC and APC states of 0.55 ns^-1^ has been omitted for clarity.

SAS and species population kinetics (**Figure 7A**) have been estimated with the help of the functional compartmental model of PB (**Figure 8**), which was derived from a previously published model of PB (Acuña et al., 2018; Van Stokkum et al., 2018). Fluorescence decay traces obtained from the model are compared with the experimental decays for selected wavelengths in **Figure S3**. Briefly, the model contains kinetic compartments corresponding to spectrally different pigment pools in PC and APC connected in a network topology modelled from the structure of the PB (**Figure S1**). The PB rods are composed of PC hexamers, each hexamer consisting of two types of functional compartments, PC640 (depicted in cyan in **Figure 8**) and PC650 (blue). The rods are connected to the top and two basal cylinders of the tri-cylindric core through the respective APC660 compartments. The top cylinder contains only APC660 (depicted in magenta), whereas the two bottom cylinders consist of four discs each. Half of these discs contain only APC660 (red) and equilibrate fast with the other half of these discs where there is intradisc equilibration between APC660 (orange) and APC680 (black and green).

The model resolves intra-hexamer equilibration in the rods, followed by rod-to-core energy transfer of PC650 to the APC660 in the three core cylinders. The scheme, which was established to model the kinetics at room temperature (RT) was slightly modified to distinguish between the different terminal emitter (TE) pigments. PB mutants with a deletion of the different APC680 pigments (ApcD, ApcE or ApcF, cf. **Figure S1B**) possessed slightly different steady-state emission spectra at 77 K. The differentiation between APC is confirmed here, and we establish that the timescale of the equilibration between APC675 (black) and APC680 (green) is 0.8 ns, i.e. this is the timescale of the complete equilibration in the entire PB, representing the end-to-end EET between the different TE pigments.

The estimated SAS (**Figures 7B, D**) show maxima at the expected wavelengths, and the shapes to be expected for PC and APC pigments. The lowest-energy SAS (green) belonging to the TE APC680 shows a narrow peak around 680 nm, whereas the higher-energy TE SAS (black) displays a broader peak around 675 nm. The loss of oscillator strength in 0.8 ns (green DAS in **Figure 7C**, smaller area of the green SAS in **Figure 7B**) is attributed to a small amount of annihilation, which we estimate to be present in up to 10% of the PB. More experiments with higher time resolution and in mutants lacking particular APC680 pigments (Jallet et al., 2012) are needed to further resolve the dynamics of the different APC680 pigments in the core.

### Target analysis of the PB-PSI complex at 77 K

**Figure 9:**
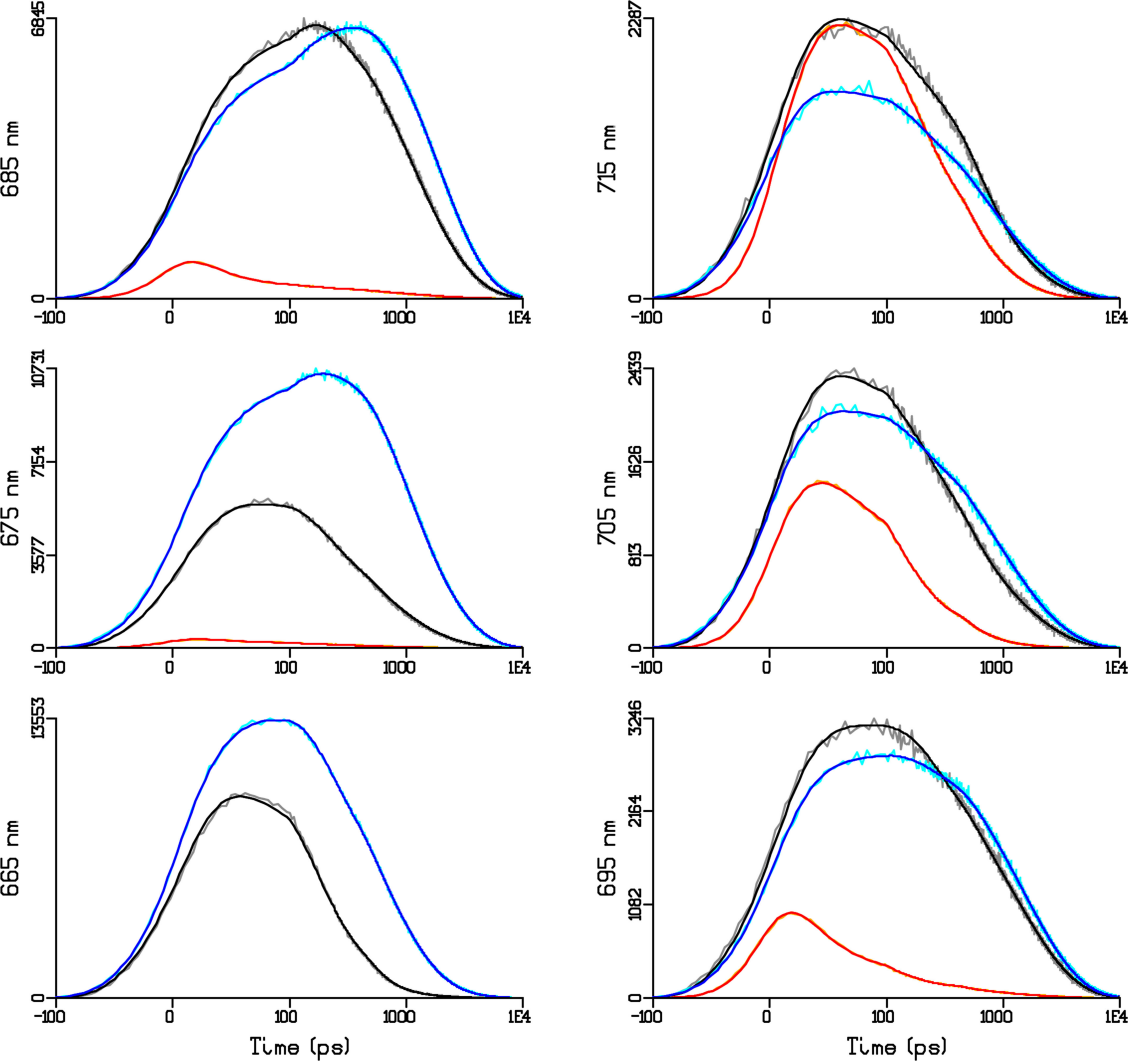
Selected time traces of the emission of the PB and the PB-PSI complex at 6 wavelengths (indicated in the ordinate label of the panels) after 580 or 440 nm excitation at 77 K. Key: 580 PB-PSI (grey), 440 PB-PSI (orange), 580 PB (cyan). Black, red and blue lines indicate the simultaneous target analysis fit. Note that the time axis is linear until 100 ps and logarithmic thereafter. Note also that each panel is scaled to its maximum. Overall rms error of the fit was 32.

Next, we present the simultaneous target analysis of the high-quality fluorescence kinetics recorded from isolated PB and from intact cells of the ΔPSII mutant of *Synechocystis* PCC 6803 at 77 K with two excitation wavelengths – 440 and 580 nm (orange and grey in **Figure 9**). ΔPSII mutant cells contain a mixture of PB-PSI complexes with different PB-PSI energy transfer rates, as well as PB that are not energetically connected to PSI.

We will combine the kinetic scheme of the PSI complex at 77 K (**Figure 5**) and the kinetic scheme of PB (**Figure 8**) (Acuña et al., 2018; Van Stokkum et al., 2018) to describe the complicated PB-PSI dynamics, cf. **Figure S4**.

The estimated fractions of the different complexes, PB-PSI complexes with fast and slow PB-PSI energy transfer rates, and non-transferring PB, are collated in **Table 1**. The time resolution of the experiments, with FWHM of the IRF ≈50 ps, is insufficient to independently estimate all microscopic rate constants. Therefore, we fixed many of the rate constants to values independently estimated *in vitro* (**Figure 8**) and *in vivo* from higher time resolution data using the same kinetic scheme for PB at 77 K (Acuña et al., 2018). The estimated fast PB–PSI energy transfer rate of 42 ns^−1^ is virtually equal to that estimated from higher time resolution data at RT. About 39% of the PB are in PB-PSI with this fast EET rate, whereas ≈10% of the PB are in PB–PSI with a slow EET rate of only 1 ns^−1^. The remaining ≈51% of the PB are non-transferring, and decay with a rate 0.54 ns^−1^. The high SNR of these TCSPC data measured until 10 ns allows us to resolve the non-transferring and the slowly transferring PB. This is in agreement with the observation at RT that the PB decay, measured until 0.5 ns, decayed more quickly than expected. Returning to the ΔPSII fluorescence emission spectrum of **Figure 2A**, the 680 nm peak in ΔPSII is attributed to ≈61% of PB that are slowly or non-transferring to PSI (**Table 1**). The ≈39% fast transferring PB then cause the still appreciable 720 nm peak of PSI Red Chl *a* (**Figure 2B**).

**Table 1:** Estimated fractions of the different complexes.

Complex\excitation	580 nm	440 nm
PB-PSI fast EET 42 ns^-1^	38.9%	40.5%
PB-PSI slow EET 0.5 ns^-1^	10.1%	5.2%
Non-transferring PB	51.0%	54.3%

**Figure 10:**
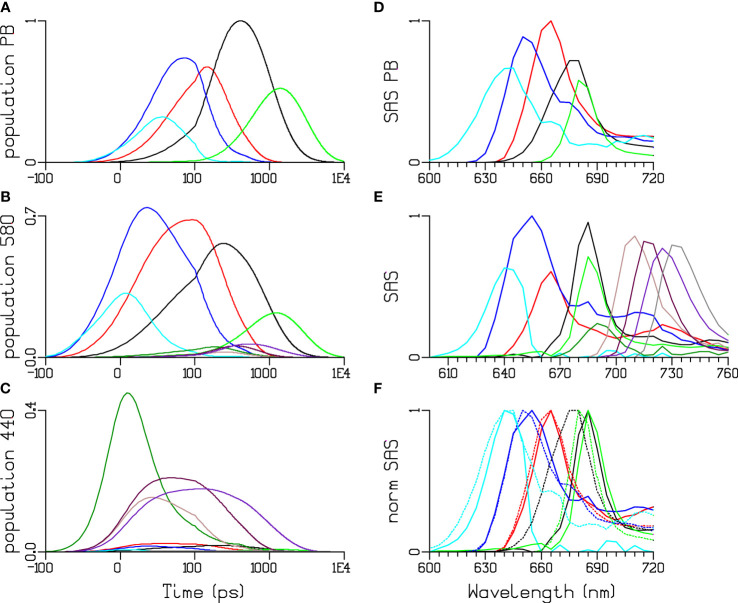
Simultaneous target analysis of PB **(A, D)** and the PB-PSI complex **(B, C, E, F)** at 77 K. Total concentrations estimated after 580 **(A, B)** or 440 **(C)** nm exc. SAS **(D, E)** and normalized SAS (F, PB dotted). Key: PC640 (cyan), PC650 (blue), APC660 (red), “APC680” (black, green), and PSI Chl *a* (dark green). Four pools of red Chl *a* have been resolved in PSI: brown, maroon, purple and grey. Note that the time axis in **(A–C)** is linear until 100 ps and logarithmic thereafter.

The quality of the target analysis fit in **Figure 9**. is excellent. Note that in the two PB–PSI experiments the relative contribution of PB and Chl *a* emission is almost opposite (cf. **Figures 10B, C** and **Figure 9**). The differences in the amplitudes of the APC660 SAS (red) can be attributed to a small amount of annihilation (**Figures 10D, E**). The shapes of the SAS from PB *in vitro* and *in vivo* (solid and dotted in **Figure 10F**) are consistent. It is only possible to resolve the dynamics and SAS of the ten different species in the simultaneous target analysis thanks to a large amount of high-signal-quality data (≈200,000 data points, cf. **Figure 9**. ) measured in different samples and under different excitation conditions.

An alternative kinetic scheme was tested wherein energy is transferred to PSI exclusively from the PC650 pigments (of the bottom cylinders) instead of APC680. The estimated EET rate constant from the PC650 (of the bottom cylinders) to PSI was 6.3 ns^−1^. However, the model failed to reproduce the experimental fluorescence kinetics of cells with 580 nm excitation, with most clearly visible differences at wavelengths 680–690 nm (**Figure S5**). The overall rms error of the fit increased from 32 to 35. Because of the misfit we must reject this alternative kinetic scheme and conclude that EET to PSI proceeds via the PB core.

## Conclusions and outlook

This work, together with the results obtained at RT (Biswas et al., manuscript in preparation), firmly establish the existence of direct EET from PBs to PSI in *Synechocystis* sp. 6803. The kinetic modelling results show that the overall trapping of energy absorbed by the PBs is rate-limited by equilibration within the PB – including both EET from the rods to the core and equilibration between the core cylinders, whereas the microscopic rate constant of EET from the terminal emitters APC680 to Chls is comparatively higher. In this respect, the “inverted” kinetics, i.e. slow equilibration within the PBs followed by rapid transfer to the reaction center and trapping, is not indifferent to the EET dynamics from PBs to PSII. Moreover, at least two distinct populations of PBs are kinetically resolved – one that transfers energy to PSI with a high quantum efficiency and comprises about 40% of the PBs in the cells, and another that is not connected to PSI at all. The existence of a fast EET route strongly suggests that the connected fraction represents PB−PSI complexes wherein the APC core of the PB specifically interacts with membrane-intrinsic PSI protein subunits for optimized pigment arrangement.

The measurements at 77 K could also confirm the existence of multiple red-shifted spectral forms of APC *in vitro* and *in vivo* – represented by APC675 and APC680 in the model. The very slow equilibration between them at 77 K suggests that they are relatively far apart of each other in different APC discs. However, it cannot be ruled out that these forms are due to spectral inhomogeneity. It also remains an open question which APC subunits (ApcD, ApcE, ApcF) contribute the most to the fast EET to PSI. This could be tested with measurements on mutants lacking particular APC pigments/subunits.

Now that we have established the energy transfer pathways in the two mutants which lack either PSI (Acuña et al., 2018) or PSII (this work), the next challenges are to establish those pathways in WT cells, and in mutants lacking particular APC680 pigments (Jallet et al., 2012).

## Data availability statement

The raw data supporting the conclusions of this article will be made available by the authors, without undue reservation.

## Author contributions

IV: Formal analysis, Methodology, Software, Validation, Visualization, Writing – original draft. PA: Conceptualization, Data curation, Formal analysis, Funding acquisition, Investigation, Validation, Visualization, Writing – original draft. AB: Data curation, Investigation, Writing – original draft. PL: Conceptualization, Formal analysis, Funding acquisition, Investigation, Project administration, Software, Supervision, Writing – original draft.
