# Supplementary material for: Energy transfer from phycobilisomes to photosystem I at 77 K
Source: Front Plant Sci. 2023 Nov 22;14:1293813. doi: 10.3389/fpls.2023.1293813 (PMC10702739; doi:10.3389/fpls.2023.1293813)
Supplement: Supplementary file 1 [file DataSheet_1.pdf]

Energy Transfer from Phycobilisomes to Photosystem I at 77 K

Ivo H.M. van Stokkum, Parveen Akhtar, Avratanu Biswas and Petar H. Lambrev

Supplementary Material

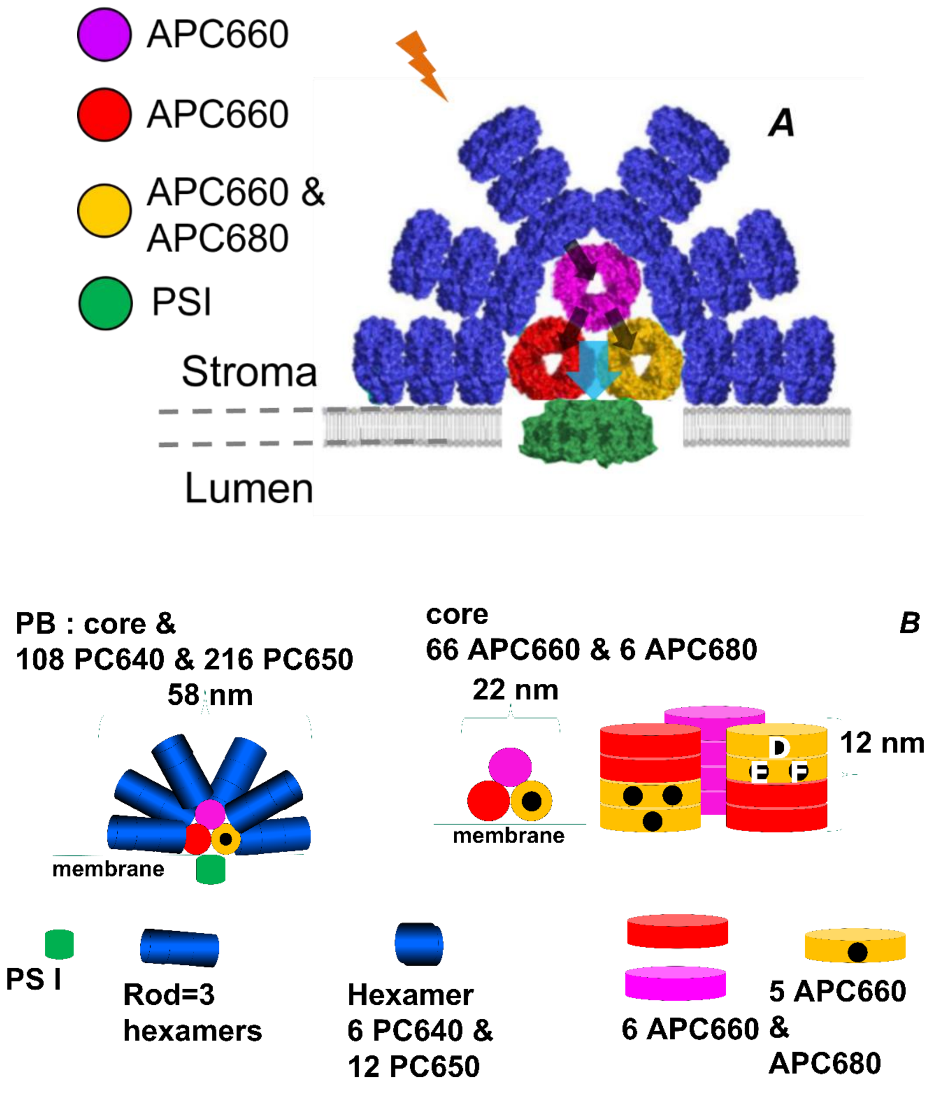

Figure S1. (A) Cartoon in side-view of a PB-PSI complex in the Synechocystis ΔPSII mutant. Key: blue, rods consisting of three hexamers; top and basal core cylinders respectively in magenta, red and orange; green, PS I trimer. Dark arrows represent intra-PB EET; the cyan arrow represents EET from the PB core to PSI. Panel B depicts the location of the different pigments in the structure. The letters D,E,F indicate the three different APC680 pigments. The approximate length for each subunit is based on (Arteni et al., 2009). Figure adapted from (Liu, 2016, Acuña et al., 2018).

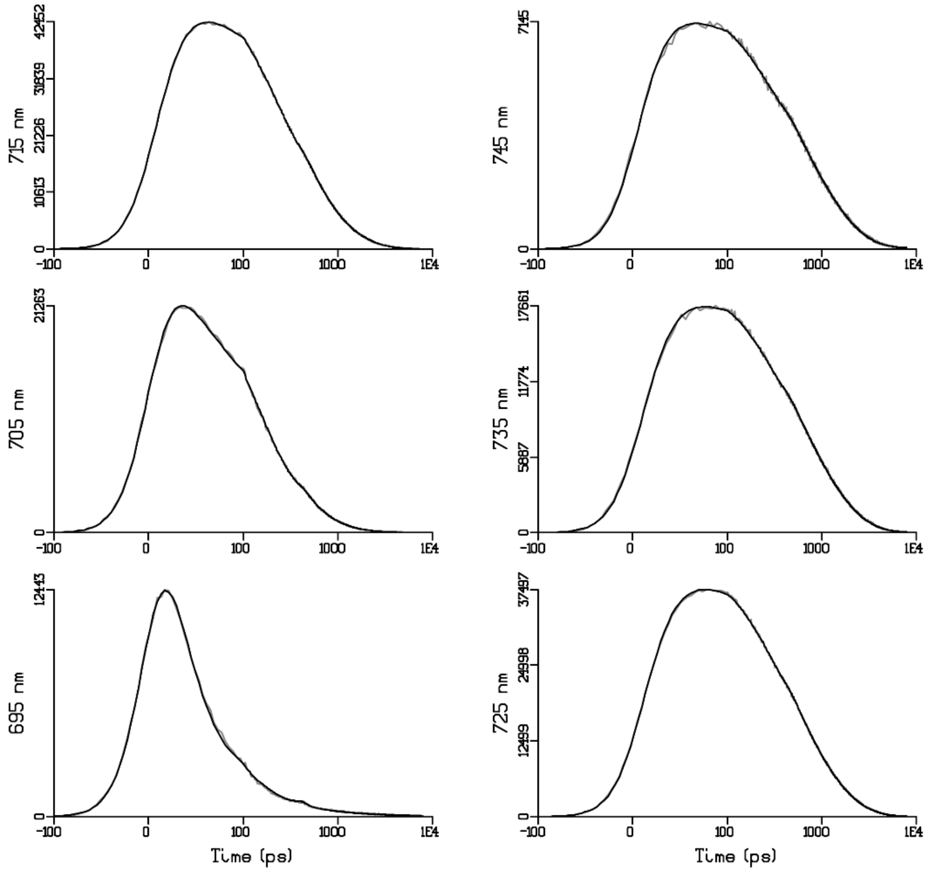

Figure S2. Selected time traces of the PSI emission at 6 wavelengths (indicated in the ordinate label of the panels) after 440 nm excitation at 77 K. Grey and black lines indicate the data and the target analysis fit, respectively. Note that the time axis is linear until 100 ps and logarithmic thereafter. Note also that each panel is scaled to its maximum. Overall RMS error of the fit was 45.

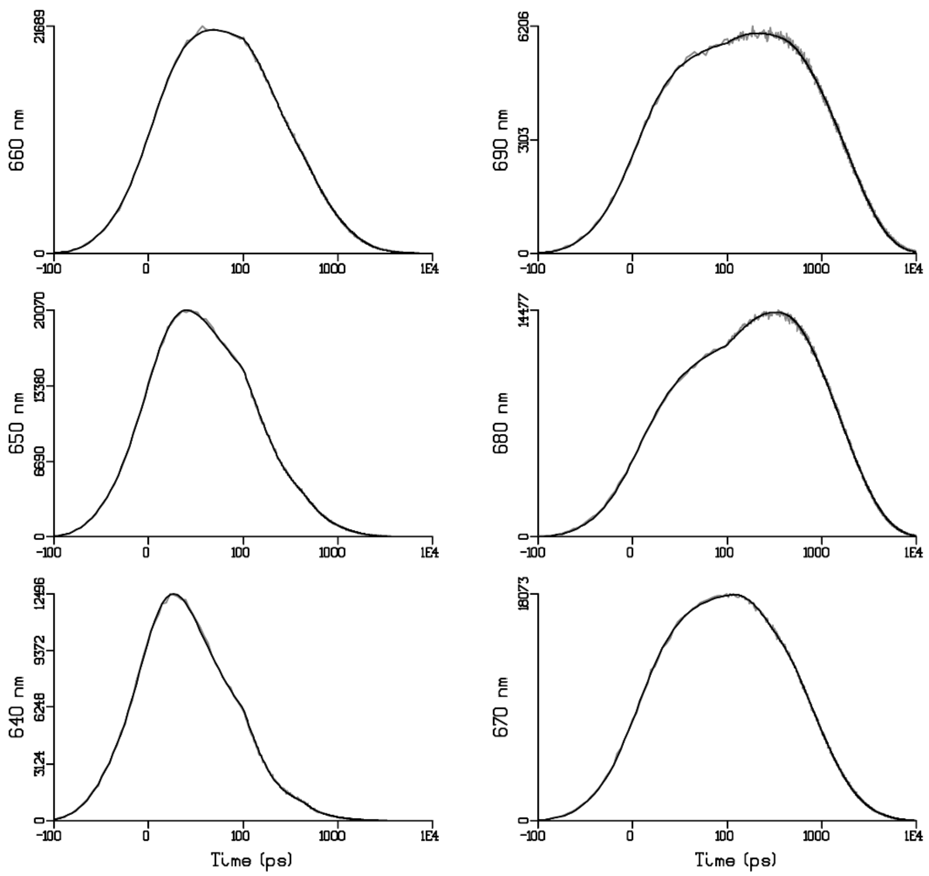

Figure S3. Selected time traces of the PB emission at 6 wavelengths (indicated in the ordinate label of the panels) after 580 nm excitation at 77 K. Grey and black lines indicate the data and the target analysis fit, respectively. Note that the time axis is linear until 100 ps and logarithmic thereafter. Note also that each panel is scaled to its maximum. Overall RMS error of the fit was 32.

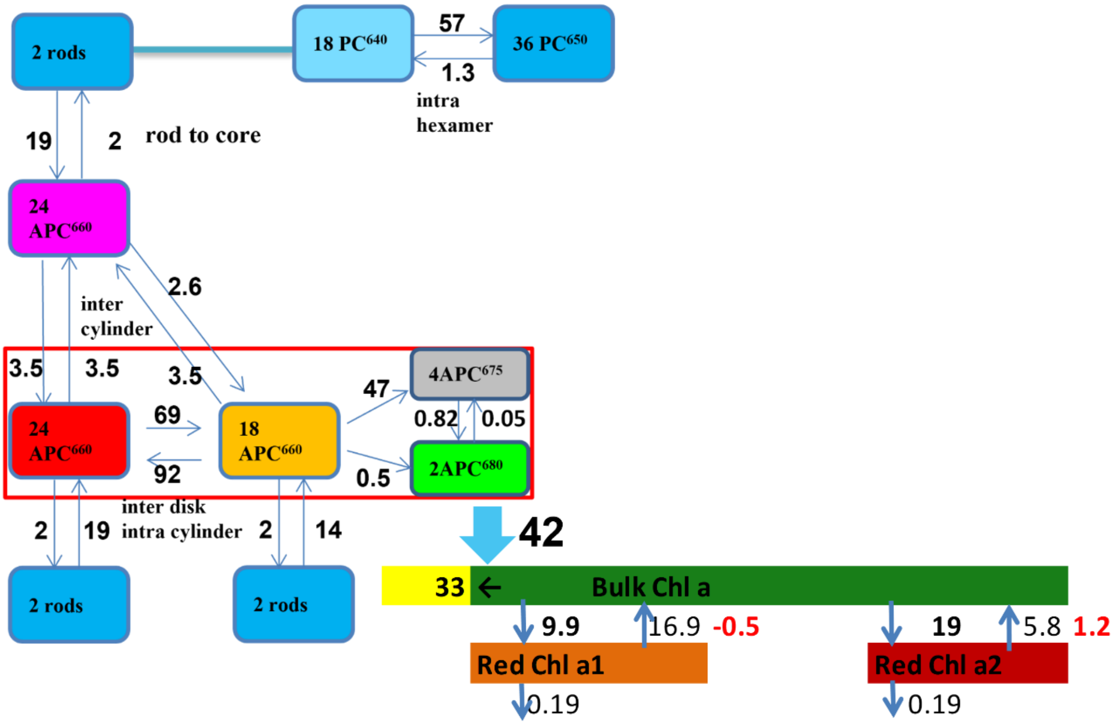

Figure S4. Minimal kinetic scheme of the PB–PSI complex at 77 K with microscopic rate constants in ns^{-1}. The thick cyan arrow represents EET (42 ns^{-1}) from the PB core to PSI. Functional compartmental model of PB, with a zoom out of a rod consisting of three lumped hexamers in the upper right. The rods contain PC640 (cyan) and PC650 (blue). The magenta APC660 compartment represents the top cylinder. The red rectangle indicates the two basal cylinders, with APC660 (orange) and APC680 (black and green) in 4 discs, and APC660 (red) in 4 other discs. The common rate constant for excited PC and APC states of 0.54 ns^{-1} has been omitted for clarity. The dark green compartment represents the Bulk PSI Chl *a* including the reaction center, which is equilibrium with two pools of Red Chl *a* (brown, maroon). The free energy differences relative to the Bulk PSI Chl *a* are indicated in red (in units of k_BT). The rate of trapping (33 ns^{-1}) is yellow highlighted.

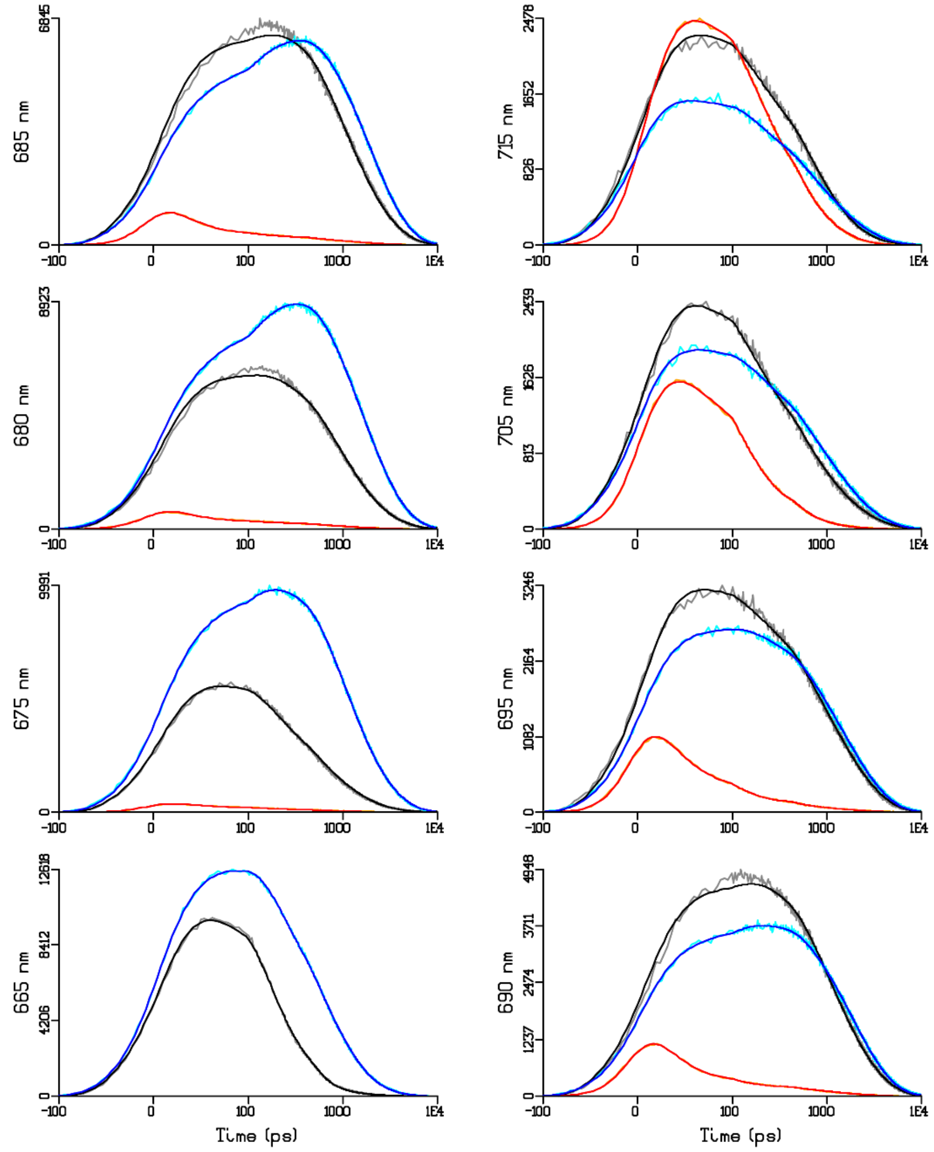

Figure S5. Selected time traces of the emission of the PB and the PB-PSI complex at 8 wavelengths (indicated in the ordinate label of the panels) after 580 or 440 nm excitation at 77 K. Key: 580 nm PB-PSI (grey), 440 nm PB-PSI (orange), 580 nm PB (cyan). Black, red and blue lines indicate the simultaneous **alternative** target analysis fit with EET to PSI from the PC650 instead of the APC680. Note that the time axis is linear until 100 ps and logarithmic thereafter. Note also that each panel is scaled to its maximum. Overall rms error of the fit was 35. Note that a clear misfit of 580 nm PB-PSI (grey) is visible at 680, 685 and 690 nm.
